# Radiofrequency ablation versus percutaneous ethanol injection for hepatocellular carcinoma: a meta-analysis of randomized controlled trials

**DOI:** 10.1186/s12957-015-0516-7

**Published:** 2015-03-08

**Authors:** Biao Yang, Rui-yu Zan, Shi-yu Wang, Xiang-lian Li, Mao-ling Wei, Wen-hao Guo, Xin You, Jing Li, Zheng-yin Liao

**Affiliations:** Department of Abdominal Oncology, West China Hospital, West China Medical School, Sichuan University, No. 17 Renming Road, Chengdu, 610000 People’s Republic of China; Department of Radiology, Shanghai Children’s Medical Center, Shanghai Jiao Tong University School of Medicine, Shanghai, 200025 China; Chinese Evidence-Based Medicine Centre, West China Hospital, West China Medical School, Sichuan University, No. 17 Renming Road, Chengdu, 610000 China

**Keywords:** Hepatocellular carcinoma, Radiofrequency ablation, Ethanol injection, Meta-analysis

## Abstract

**Background:**

Radiofrequency ablation (RFA) and percutaneous ethanol injection (PEI) are treatment methods for patients with early-stage hepatocellular carcinoma (HCC) who are not suitable for surgery. Although some reports indicate that RFA is better than PEI, results from previous reviews and analyses are inconsistent. Therefore, this meta-analysis was performed to more thoroughly evaluate the effects of these treatments in patients with HCC.

**Methods:**

A literature search was conducted using the Excerpta Medica dataBASE, PubMed, the Cochrane Library, the American Society of Clinical Oncology database, the China National Knowledge Infrastructure database, the Wanfang database, the Chinese Biomedical Literature Database, and the Chongqing VIP database without language limitations. The primary outcome evaluated was overall survival, and secondary outcomes included complete response and local recurrence. Comparisons were made between Asian and European studies.

**Results:**

Total pooled and subgroup analyses of Asian studies that included selection biases revealed that RFA is superior to PEI with respect to overall survival (hazard ratio (HR), 0.54; 95% confidence interval (CI), 0.37 to 0.80; *P* < 0.01) and complete response (relative risk (RR), 1.10; 95% CI 1.03 to 1.18; *P* < 0.01). However, no significant difference was observed between RFA and PEI in the European studies. In Asian studies, RFA was associated with a lower local recurrence rate than PEI at 1 year (RR, 0.44; 95% CI 0.20 to 0.95; *P* < 0.05) and 3 years (RR, 0.35; 95% CI 0.22 to 0.55; *P* < 0.01). However, local recurrence was significantly lower after only 3 years in European studies (RR, 0.50; 95% CI 0.32 to 0.78; *P* < 0.05).

**Conclusions:**

RFA was only superior to PEI in Asian studies that included selection bias. Thus, there is insufficient evidence to support the idea that RFA is superior to PEI for patients with cirrhotic HCC. Additional large-scale, multicenter, randomized controlled trials that control for selection bias are needed to fully elucidate the optimal treatment method for HCC.

## Background

Hepatocellular carcinoma (HCC) is the sixth most common cancer and the third most frequent cause of cancer-related deaths worldwide [1]. HCC is a global problem, and its incidence is increasing in both the United States and Europe [2-5]. Surgical resection and liver transplantation are effective for the treatment of HCC [6,7]. Nevertheless, the surgical options for HCC are often limited due to concurrent hepatic cirrhosis, poor liver function, or multiple lesions [6,8-10]. Unfortunately, liver transplantation plays a very small role in the treatment of HCC because of ethical problems and a shortage of liver donors [11,12]. Thus, various nonsurgical treatment methods have been developed, including transcatheter arterial chemoembolization, percutaneous ethanol injection (PEI), radiofrequency ablation (RFA), and percutaneous acid injection.

PEI was the first percutaneous technique introduced to clinical practice [8,13]. It is a simple, inexpensive, safe, effective technique with a low complication rate and can achieve complete necrosis of small HCC lesions [14-17]. Therefore, in 2001, the European Association for the Study of the Liver recommended PEI as the standard percutaneous treatment for early-stage, nonsurgical HCC [6]. However, PEI has some limitations, including the need for multiple treatment sessions and a prolonged treatment time [6].

In 1999, RFA was first conducted for the treatment of HCC in Japan [18]. Its advantages over PEI include ease of performance, effectiveness similar to that of surgical resection, high safety, and low invasiveness [19-22]. The American Association for the Study of Liver Diseases published guidelines in 2005 (updated in 2010) indicating that RFA is the first-choice procedure for patients with HCC, especially patients with tumors that are ≤3 cm in diameter and comprise one to three nodules. However, PEI still plays an important role in the local treatment of early-stage HCC [6,23]. Despite the advantages of RFA, complete ablation remains difficult to achieve in some specific liver sites; RFA is also more expensive than PEI [21,24].

To the best of our knowledge, four Asian randomized controlled trials (RCTs) have indicated that RFA is superior to PEI in terms of overall survival (OS), complete response (CR), and local recurrence (LR) [25-28]. However, these trials may have included selection bias, as trials performed in Europe have shown no significant differences between RFA and PEI with respect to OS [16,29-32]. The most effective treatment strategy for patients with unresectable HCC thus remains controversial. The present meta-analysis was performed to compare the effects of RFA and PEI for the treatment of HCC.

## Methods

### Search strategy

The following databases were searched for all published and unpublished RCTs with no language restrictions: Excerpta Medica dataBASE (EMBASE), PubMed, Cochrane Central Register of Controlled Trials, Cochrane Methodology Register, Cochrane Database of Systematic Reviews, Cochrane Library, American Society of Clinical Oncology database (ASCO), China National Knowledge Infrastructure database (CNKI), Chinese Biomedical Literature (CBM) Database, Wanfang database, and the Chongqing VIP database (CQVIP). Combinations of the following terms were used in the search: medical subject headings = liver neoplasms, catheter ablation, ethanol, and injections intraregional; free text terms = hepatocellular carcinoma, liver cancer, liver tumor, radiofrequency ablation, RFA, ethanol injection, alcohol injection, and PEI. We also performed a supplementary literature search through Google Scholar and some leading journals. The publication cutoff date was 14 March 2014. Each search strategy was determined after numerous pre-searches. The reference lists of all included studies were examined for relevant publications.

### Eligibility criteria

All procedures were performed in compliance with the Declaration of Helsinki. This study was also approved by the Committee on the Ethics West China Hospital. The inclusion criteria were as follows: (i) studies including patients with unresectable lesions, Child-Pugh class A or B liver function, and inclusion of both an RFA and PEI group (in studies comparing two or more arms); (ii) RFA as the intervention and PEI as the comparator; (iii) inclusion of data related to OS (percentage of patients who survived for a defined period of time after treatment), and at least one of the following secondary outcomes: LR (percentage of patients with newly detected liver tumors in the posttreatment follow-up period), CR (lower incidence of liver tumors posttreatment), and treatment-related adverse events or complications; and (iv) RCT was the study design.

The exclusion criteria were as follows: (i) phase 2 RCTs, (ii) lesions >5 cm in diameter (ablation techniques alone are less effective in larger tumors [33]), and (iii) history of surgical treatment for HCC.

### Assessment of study quality

We used the Cochrane Collaboration handbook to assess the risk of bias in all included studies [34]. The quality items assessed included sequence generation, allocation concealment, baseline imbalance, double-blinding, incomplete outcome data, early stopping, and selective outcome reporting. For individual studies, each criterion was assigned a label of ‘yes,’ ‘unclear,’ or ‘no’ to estimate the risk of bias. Two reviewers (Y.B. and W.S.Y.) performed the quality assessment. Any discrepancies were resolved by an intercessor (Z.R.Y.).

### Data extraction

The two reviewers (Y.B. and W.S.Y.) independently extracted the following parameters from all included studies: basic information of the studies (authors, publication year, duration of study, region in which the study was performed, and randomization method), patient characteristics (age, sex, number and size of tumors, number of patients with hepatitis B and C virus infection, and liver function), information on the intervention and comparator evaluated in the study, and clinical outcomes (OS, LR, CR, and complications). Most original data were extracted directly from the trials, but some data regarding OS and LR were extracted from the published study curves using the software Engauge Digitizer (version 4.1) described by Parmar [35] and calculated using a Microsoft Excel spreadsheet provided by Tierney *et al.* [36].

### Outcome measures

In this meta-analysis, the primary outcome was OS. The secondary outcomes were LR, CR, and the main treatment-related complications that occurred during the follow-up period.

### Statistical analysis

The hazard ratio (HR) and 95% confidence interval (CI) of the OS were calculated for each trial. Similarly, the risk ratio (RR) and 95% CI of the LR and CR were determined. Subgroup analyses were performed to explore important clinical differences between European and Asian trials. Both random and fixed effects models were utilized [37,38]. *I*^2^ and *χ*^2^ statistics of the HR were used to evaluate endpoint heterogeneity; studies with a *P* < 0.1 and *I*^2^ > 50% were considered invalid, and the random effects model was used. Otherwise, the data were pooled using the fixed effects model. The significance of the pooled RR was determined by the *Z*-test, and *P* < 0.05 was considered statistically significant. All data obtained in this meta-analysis were pooled and analyzed using the software program Review Manager (version 5.0). Microsoft Excel 2010 was used to calculate the data. The guidelines detailed in this review are described in the Cochrane handbook [34].

## Results

### Study selection

**Figure 1:**
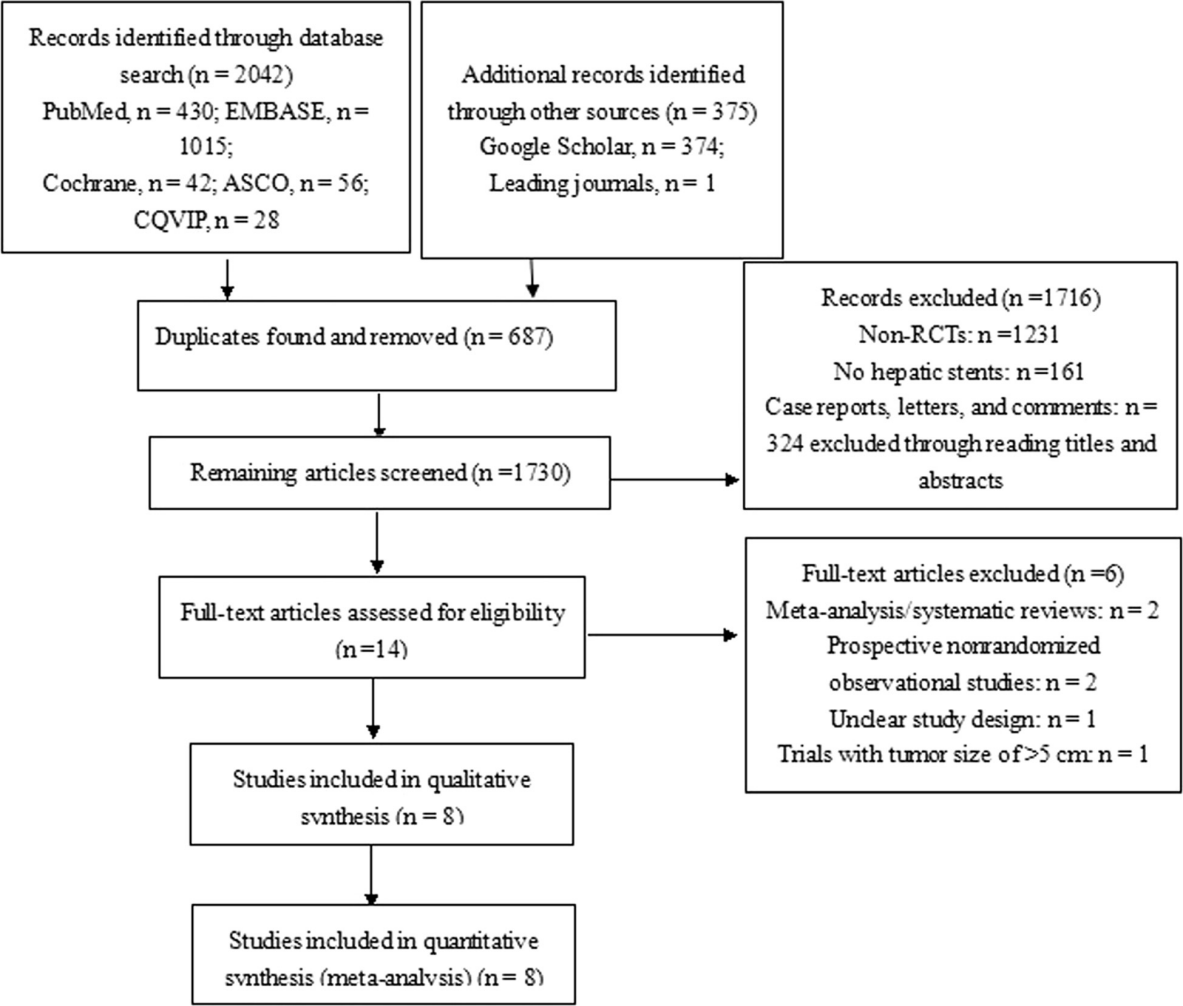
**Study flow chart.** ASCO, American Society of Clinical Oncology; CNKI, China National Knowledge Infrastructure; CBM, Chinese Biomedical Literature Database; CQVIP, Chongqing VIP database; EMBASE, Excerpta Medica dataBASE.

In total, 2,417 citations were identified in the search of the electronic databases (Figure 1); 687 duplicates were excluded, and 1,730 papers remained. We excluded 1,716 citations based on reading the title and abstract in our initial screening, and 14 full-text articles were read for further assessment. Two papers reported prospective studies without additional details. One prospective nonrandomized study was excluded after obtaining information via email from the author. However, we failed to contact the corresponding author of another prospective nonrandomized study and thus excluded this paper because of its unclear study design. The remaining papers were evaluated, and several more were excluded for the reasons listed in Figure 1. Finally, eight RCTs met all eligibility criteria and were included in this meta-analysis [25-28,30,31,39,40]. Three RCTs were conducted in Italy and were thus grouped as European studies [30,31,40], four originated from Asia (Taiwan, *n* = 2 [25,26]; Japan, *n* = 1 [27]; and China, *n* = 1 [28]), and one study from Egypt was considered as an African study [39]. Although the two studies from Taiwan were performed in the same institution within 2 years, the corresponding author indicated that the randomized patients in these two studies did not overlap.

### Characteristics of included studies

In total, 1,130 patients were included among all selected studies; 561 underwent RFA, and 569 underwent PEI. The RFA and PEI groups contained 319 and 320 patients with Child-Pugh class A liver function, respectively. The average number of treatment sessions per tumor ranged from 1.1 to 8.0. The male to female ratio was 1.97:1.00. The mean tumor size ranged from 2.25 ± 0.45 to 2.8 ± 0.8 cm. A total of 758 patients had a single lesion (RF, *n* = 381; PEI, *n* = 377). The patients’ baseline characteristics are presented in Table 1, and the details of the study endpoints are summarized in Table 2. Details on the quality of each included study are presented in Table 3. A total of 466 complications or adverse events occurred among all patients in all studies (RFA, *n* = 247; PEI, *n* = 219) (Table 4).

**Table 1:** **Study and patient characteristics of each randomized controlled trial**

**First author, year**	**Duration**	**Region**	**Randomization**	**Tx (** ***n*** **)**	**Age, years**	**Sex,** ***n*** **(M/F)**	**Child-Pugh,** ***n*** **(A/B)**	**Infection,** ***n*** **(HBV/HCV/others)**	**Tumors,** ***n*** **(1/>2)**	**Ratio of tumor,** ***n*** **(≤3/> 3 cm)**	**Tumor size, cm**	**Albumin, g/dL**	**Volume per session, mL**	**Number of sessions**	**Follow-up, months**	**Jadad score**
Lencioni, 2003	April 2000 to April 2002	Italy	Computer	RFA (52)	6 ± 6.0	36/16	45/7	6/22/24	40/12	46/6	2.8 ± 0.6	4.1 ± 0.7	NA	1.1 ± 0.5	22.9 ± 9.4	4
				PEI (50)	6 ± 7.4	30/20	35/15	9/20/21	31/19	42/8	2.8 ± 0.8	3.7 ± 0.6	NA	5.4 ± 1.6	22.4 ± 8.6	
Lin, 2004	April 2000 to April 2002	Taiwan	Computer	RFA (52)	62 ± 11	35/17	41/11	35/16/1	38/14	37/15	2.9 ± 0.8	3.7 ± 0.8	NA	1.6 ± 0.4	24.5 ± 11.3	4
				PEI (52)	59 ± 10	34/18	39/12	37/14/1	40/12	38/14	2.8 ± 0.8	3.8 ± 1.0	4.5 (2.0–10.0)	6.5 ± 1.6	23.8 ± 10.4	
Lin, 2005	April 2000 to April 2003	Taiwan	Computer	RFA (62)	61 ± 10	40/22	46/16	41/20/1	49/13	62/0	2.5 ± 1.0	4.0 ± 0.6		1.3 ± 0.3	28 ± 12	4
				PEI (62)	60 ± 8	39/23	47/15	42/19/1	49/13	62/0	2.3 ± 0.8	3.9 ± 0.4	4.8 (2.0–10.4)	4.9 ± 1.3	26 ± 13	
Shiina, 2005	April 1999 to January 2001	Japan	Computer	RFA (118)	44/71 (≤65/> 65)	79/39	85/33	18/90/10	72/46	118/0	NA	60/58 (≤3.5/> 3.5)	2–8	2.1 ± 1.3	37	4
				PEI (114)	41/73 (≥65/> 65)	87/27	85/29	11/98/5	60/54	114/0	NA	49/65 (≤3.5/> 3.5)	NA	6.4 ± 2.6	35	
Brunello, 2008	January 2001 to September 2004	Italy	Computer	RFA (70)	69.0 ± 7.7	43/27	39/31	6/44/20	54/16	70/0	2.42 ± 0.49	3.45 ± 0.69	2–20	NA	26.1	4
				PEI (69)	70.3 ± 8.1	49/20	39/30	0/47/22	54/15	69/0	2.25 ± 0.54	3.42 ± 0.55	NA	NA	25.3	
Giorgio, 2011	January 2005 to January 2010	Italy	RNG	RFA (128)	70 ± 2	105/37	70/72	61/81/0	128	128/0	2.34 ± 0.45	3.37 ± 0.87	8.7 (4.0–20.0)	5	22	4
				PEI (143)	72 ± 6	102/41	75/68	56/87/0	143	143/0	2.27 ± 0.48	3.41 ± 0.92	NA	8	22	
Wang, 2011	January 2001 to March 2009	China	NA	RFA (49)	43	35/14	NA	NA	NA	NA	2.4 ± 1.2	NA	NA	NA	11.2 ± 1.3	2
				PEI (49)	45	36/13	NA	NA	NA	NA	2.3 ± 1.4	NA		NA	11.2 ± 1.3	
Azab, 2011	2005 to 2008	Egypt	NA	RFA (30)	NA	NA	NA	NA	NA	16/17	NA	NA	3–14	1.45	NA	3
				PEI (30)	NA	NA	NA	NA	NA	16/16	NA	NA		7.68	NA	

HBV, hepatitis B virus; HCV, hepatitis C virus; NA, not available; PEI, percutaneous ethanol injection; RFA, radiofrequency ablation; RNG, random number generator; Tx, treatment.

Data are presented as mean ± standard deviation or mean (range).

**Table 2:** **Efficacy of radiofrequency ablation versus percutaneous ethanol injection for treatment of hepatocellular carcinoma**

**First author, year**	**Treatment (** ***n*** **)**	**Complete response (%)**	**Overall survival (%)**					**Local recurrence (%)**				
			**1-year**	**2-year**	**3-year**	**4-year**	**5-year**	**1-year**	**2-year**	**3-year**	**4-year**	**5-year**
Lencioni, 2003	RFA (52)	91.0	100.0	98.0	NA	NA	NA	2	4	21+	NA	NA
	PEI (50)	82.0	96.0	88.0	NA	NA	NA	17.0	38.0	59.0+	NA	NA
Lin, 2004	RFA (52)	96.0	90.0	82.0	74.0	NA	NA	12.0	18.0	18.0	NA	NA
	PEI (52)	88.0	85.0	61.0	50.0	NA	NA	23.0	45.0	45.0	NA	NA
Lin, 2005	RFA (62)	96.1	93.0	81.0	74.0	NA	NA	10.0	14.0	14.0	NA	NA
	PEI (62)	88.1	88.0	66.0	51.0	NA	NA	16.0	34.0	34.0	NA	NA
Shiina, 2005	RFA (118)	100.0	96.0+	91.0+	81.0+	74.0	NA	1.0+	1.7+	1.7+	1.7+	NA
	PEI (114)	100.0	93.0+	81.0+	66.0+	57.0	NA	9.0+	11.0+	11.0+	11.0+	NA
Brunello, 2008	RFA (70)	95.7	94.0+	77.0+	59.0+	44.0+	NA	NA	NA	NA	NA	NA
	PEI (69)	65.6	87.0+	74.0+	56.0+	42.0+	NA	NA	NA	NA	NA	NA
Giorgio, 2011	RFA (128)	100.0	95.0	90.0	83.0	73.0	70.0	4.1	5.7	7.8	8.9	11.7
	PEI (143)	100.0	95.0	83.0	78.0	70.0	68.0	5.2	6.7	9.4	11.5	12.8
Wang, 2011	RFA (49)	93.8	95.9	91.8	NA	NA	NA	NA	NA	NA	NA	NA
	PEI (49)	77.5	85.7	77.5	NA	NA	NA	NA	NA	NA	NA	NA
Azab, 2011	RFA (30)	85.0	90.0	NA	NA	NA	NA	NA	NA	NA	NA	NA
	PEI (30)	75.0	83.3	NA	NA	NA	NA	NA	NA	NA	NA	NA

NA, not available; PEI, percutaneous ethanol injection; RFA, radiofrequency ablation.

+, data extracted from survival curves.

**Table 3:** **Risk of bias in the trials included in the present meta-analysis**

**Bias**	**Lencioni, 2003**	**Lin, 2004**	**Lin, 2005**	**Shiina, 2005**	**Brunello, 2008**	**Giorgio, 2011**	**Wang, 2011**	**Azab, 2011**
Sequence generation	+	+	+	+	+	+	?	?
Allocation concealment	?	?	?	?	+	+	?	?
Double-blinding	−	−	−	−	−	−	−	−
Incomplete outcome data	−	+	−	+	−	−	−	−
Selective outcome reporting	−	−	−	−	+	−	−	−
Other sources of bias	−	+	+	−	−	−	−	+
Early stopping	?	−	−	−	+	−	−	−
Baseline imbalance	−	−	−	−	−	−	−	−

+, yes; −, no; ?, unclear.

**Table 4:** **Major complications associated with radiofrequency ablation and percutaneous ethanol injection**

**Complication**	**RFA (** ***n*** **= 247)**	**PEI (** ***n*** **= 219)**
Fever	58	52
Skin burns	6	0
Ascites	59	48
Jaundice	7	6
Liver abscess	0	2
Gastric bleeding	1	1
Pain	98	100
Hydrothorax	8	0
Hemothorax	4	1
Subcapsular hematoma	1	0
Portal venous thrombosis	5	9

### Overall survival

**Figure 2:**
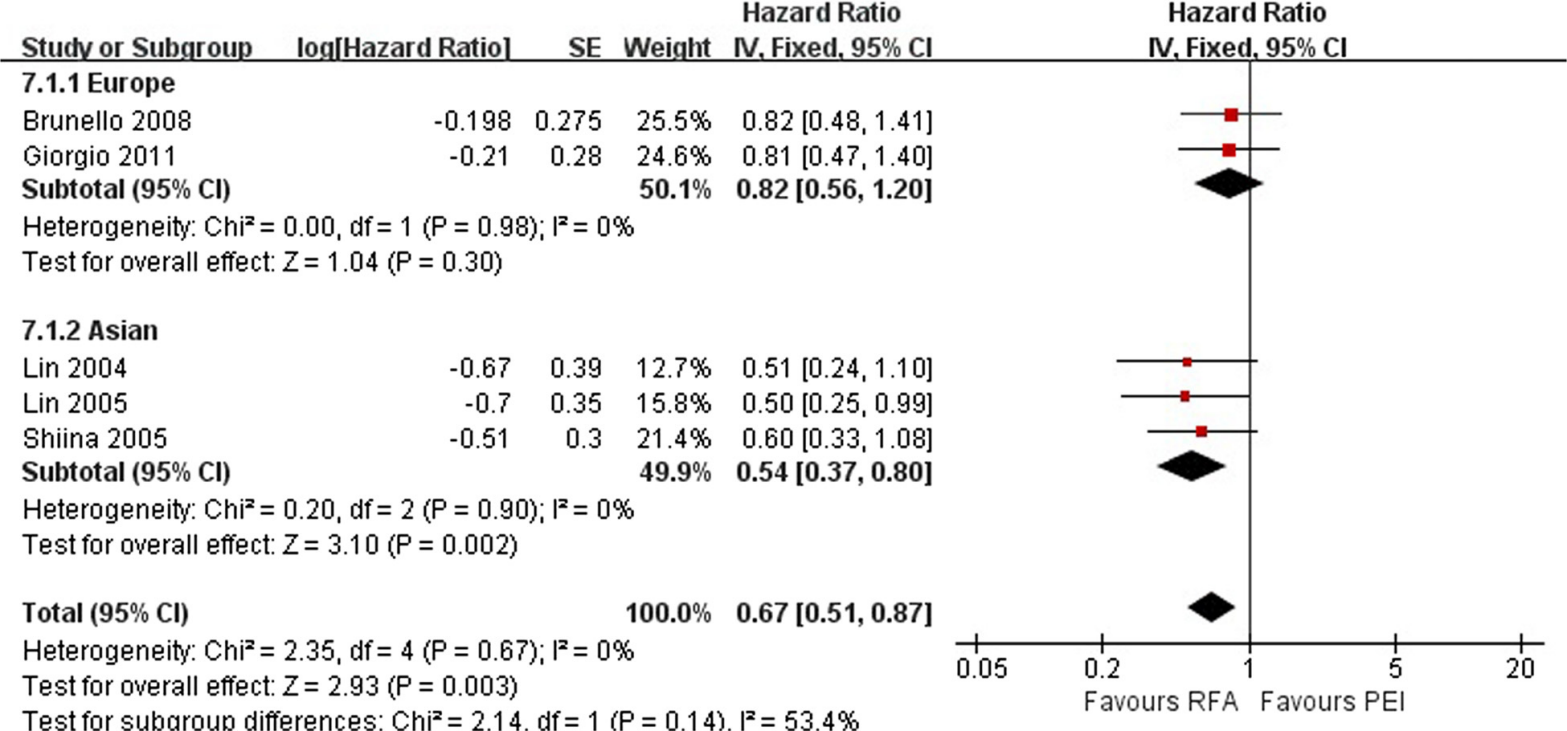
**Comparison of overall survival between radiofrequency ablation and percutaneous ethanol injection for hepatocellular carcinoma (fixed effects model).** CI, confidence interval; HR, hazard ratio; PEI, percutaneous ethanol injection; RFA, radiofrequency ablation; SE, standard error.

Sensitivity analysis did not change the significance of the results in the Asian studies. A fixed effects model was used because of a lack of significant heterogeneity as follows: Asia, *I*^2^ = 0%, *P* = 0.98; Europe, *I*^2^ = 0%, *P* = 0.90; and total, *I*^2^ = 0%, *P* = 0.67 (Figure 2). Total effect of comparison of RFA with PEI indicated that RFA was associated with better OS rates than PEI (HR, 0.67; 95% CI 0.51 to 0.87; *P* < 0.01). In the Asian studies, RFA exhibited better performance than PEI in terms of OS (HR, 0.54; 95% CI: 0.37 to 0.80; *P* < 0.01). However, no statistically significant difference was observed between the two treatments in the European studies (HR, 0.82; 95% CI 0.56 to 1.20; *P* = 0.30).

### Local recurrence rate

Only five RCTs evaluated the LR because three did not report relevant data [25-27,31,40]. Only one study conducted in Europe reported the LR associated with a long-term follow-up; thus, a subgroup analysis with HR could not be performed. The RR was deemed to be an optimal indicator to obtain additional information from these original studies through comparison of RFA with PEI.

**Figure 3:**
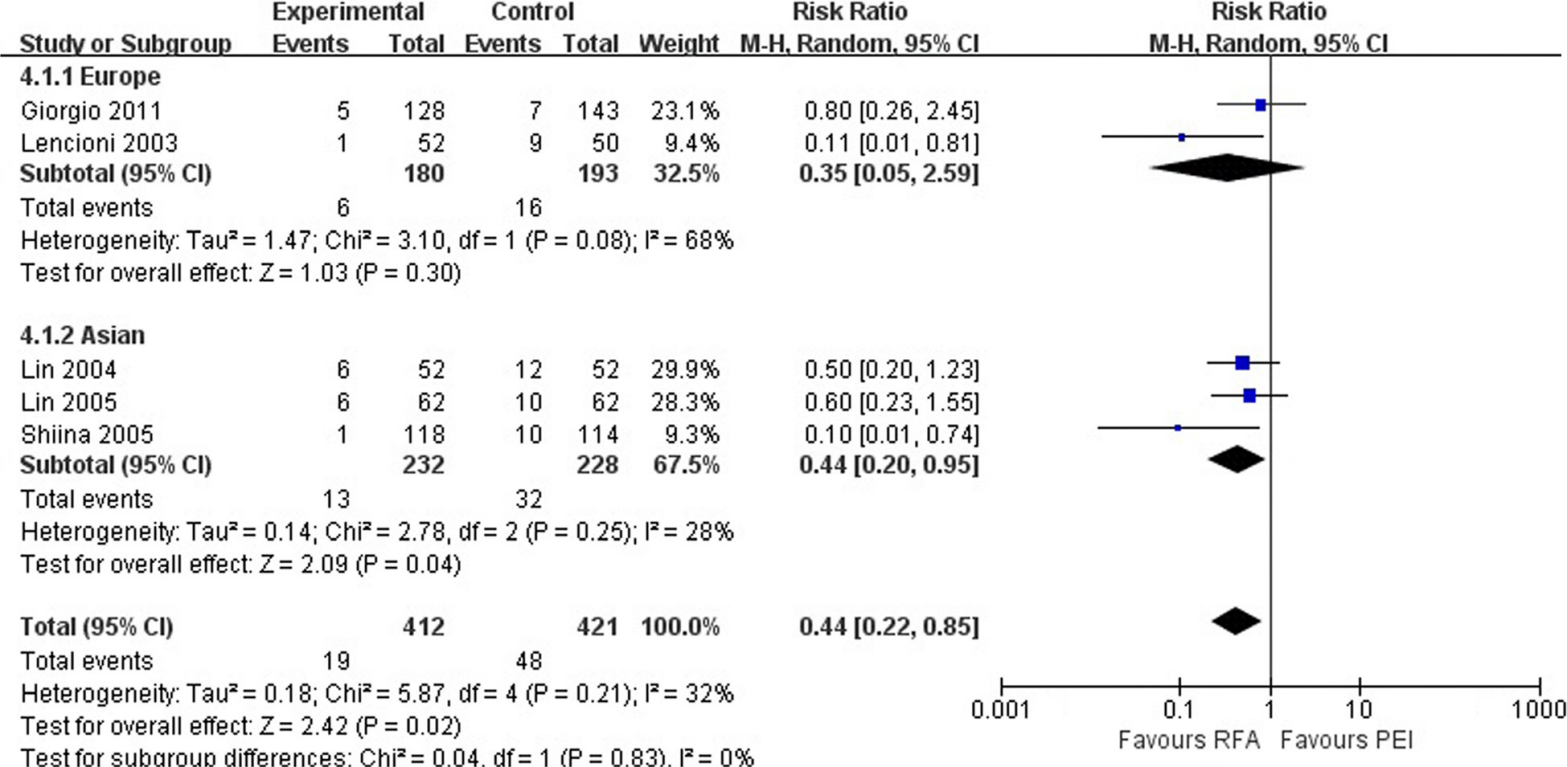
**Comparison of one-year local recurrence rates between radiofrequency ablation and percutaneous ethanol injection for hepatocellular carcinoma (random effects model).** CI, confidence interval; PEI, percutaneous ethanol injection; RFA, radiofrequency ablation; RR, risk ratio.

**Figure 4:**
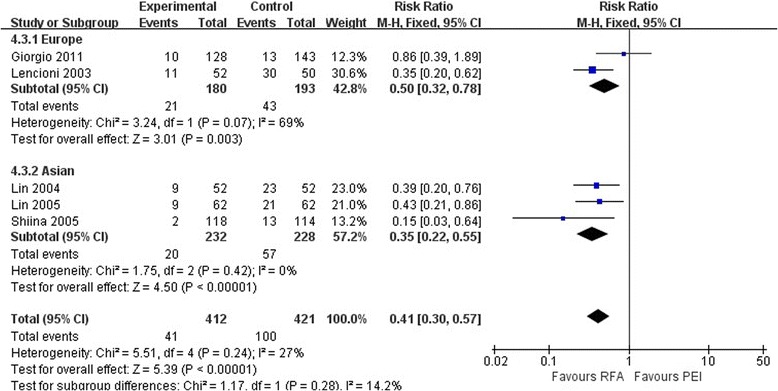
**Comparison of three-year local recurrence rates between radiofrequency ablation and percutaneous ethanol injection for hepatocellular carcinoma (random effects model).** CI, confidence interval; PEI, percutaneous ethanol injection; RFA, radiofrequency ablation; RR, risk ratio.

As shown in Figures 3 and 4, the pooled estimate of the treatment indicated that RFA was associated with a lower LR rate than was PEI at 1 year (RR, 0.44; 95% CI 0.22 to 0.85; *P* = 0.02) and 3 years (RR, 0.41; 95% CI 0.30 to 0.57; *P* < 0.01). The same condition was found in the Asian studies at 1 year (RR, 0.44; 95% CI 0.20 to 0.95; *P* = 0.04) and 3 years (RR, 0.35; 95% CI 0.22 to 0.55; *P* < 0.01). In the European studies, however, no significant differences were observed between the two treatment groups at 1 year (RR, 0.35; 95% CI 0.05 to 2.59; *P* = 0.30) or 3 years (RR, 0.50; 95% CI 0.32 to 0.78; *P* < 0.01). The European data showed heterogeneity at 1 year (*I*^2^ = 68%, *P* = 0.08) and 3 years (*I*^2^ = 69%, *P* = 0.07). Thus, the random effects models were derived.

### Complete response

All eight studies reported the CR rate after RFA or PEI, and six of them were suitable for pooling of the CR data (both RFA and PEI exhibited a 100% CR rate in the two remaining studies [27,31]).

**Figure 5:**
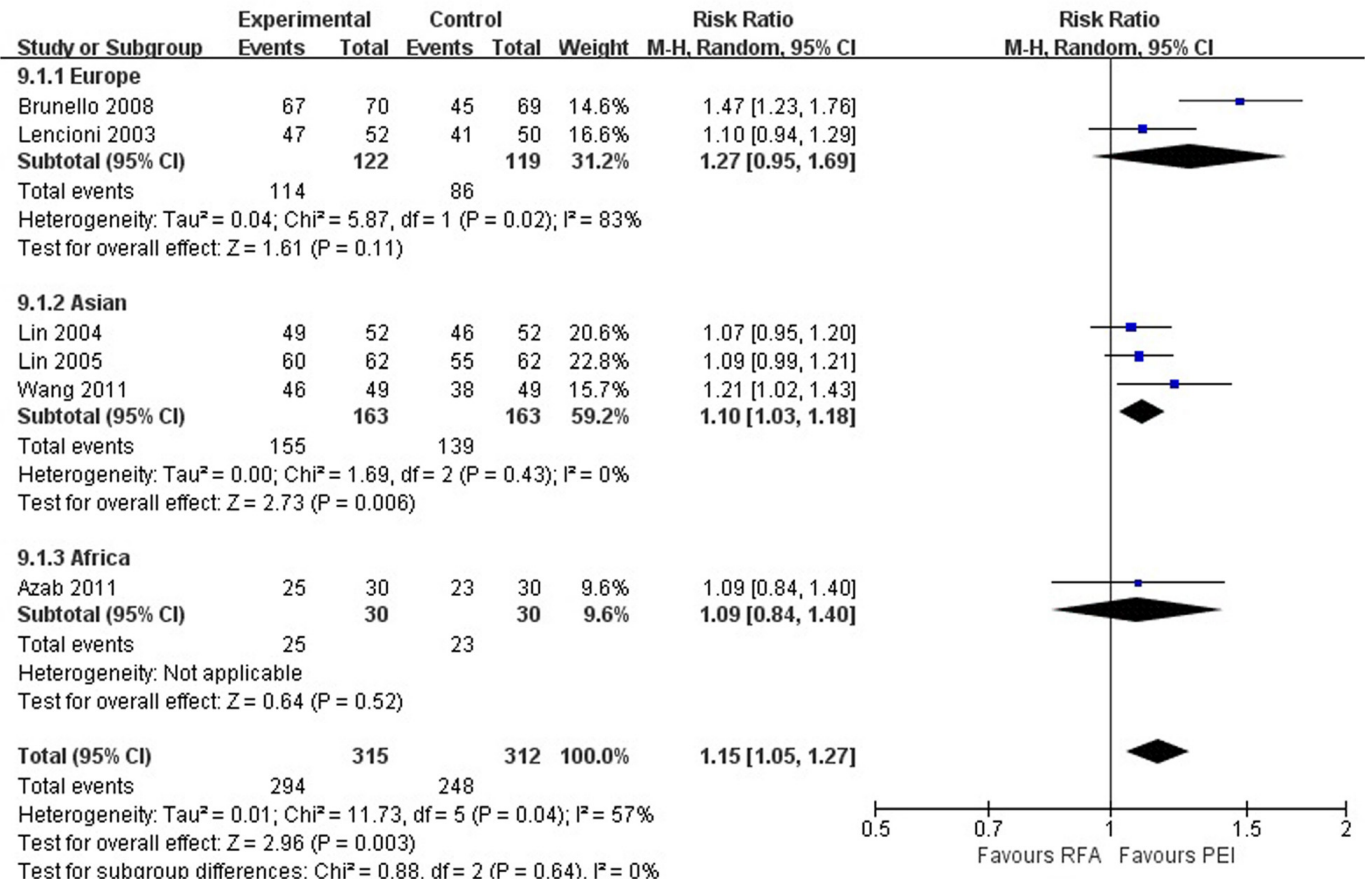
**Comparison of complete responses between radiofrequency ablation and percutaneous ethanol injection for hepatocellular carcinoma (random effects model).** CI, confidence interval; PEI, percutaneous ethanol injection; RFA, radiofrequency ablation; RR, risk ratio.

The total pooled data showed that RFA was associated with a better CR rate than PEI (RR, 1.15; 95% CI 1.05 to 1.27; *P* < 0.01) (Figure 5). A similar finding was observed in the Asian studies (RR, 1.10; 95% CI 1.03 to 1.18; *P* < 0.01). However, no significant differences were found between the two treatment groups in the European studies (RR, 1.27; 95% CI 0.95 to 1.69; *P* = 0.11) or in the African study (RR, 1.09; 95% CI 0.84 to 1.40; *P* = 0.52).

### Complications

The main adverse events in the two treatment groups are presented in Table 4. RFA was associated with more complications than was PEI (*n* = 247 and 219, respectively). The main differences between the two treatment groups involved the incidence of skin burns, hydrothorax, liver abscesses, hemothorax, and portal venous thrombosis.

### Sensitivity analysis and publication bias

**Figure 6:**
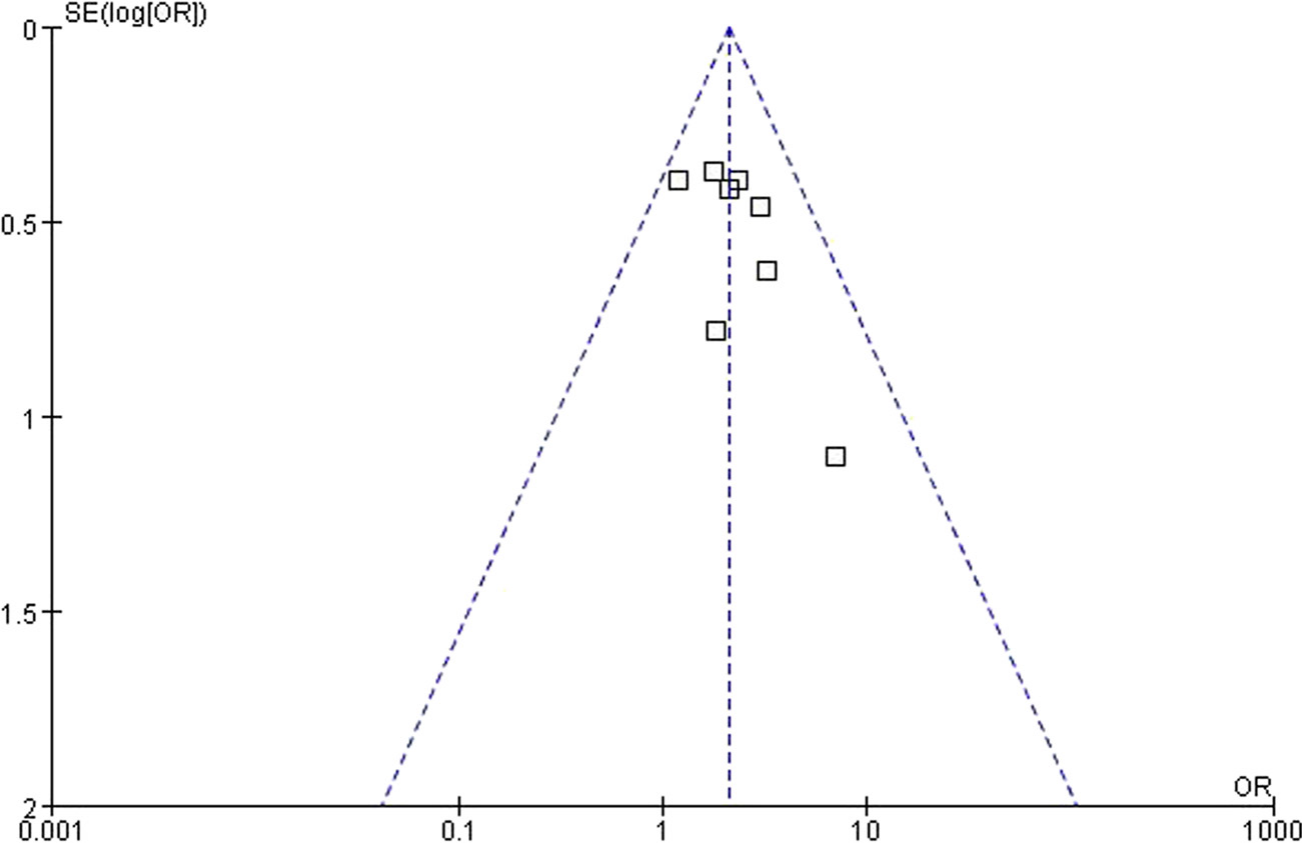
**Funnel plot for assessment of publication bias in included studies.** OR, odds ratio; SE, standard error.

We performed a one-way sensitivity analysis to evaluate the stability of the meta-analysis with respect to the Asian studies. The sensitivity analysis did not change the significance of the results in the Asian studies. A sensitivity analysis was not performed for the European trials because only two studies were available [30,31]. A funnel plot indicated that no significant publication bias was observed in any of the studies (Figure 6).

## Discussion

The results from the Asian studies included in this meta-analysis indicate that RFA is superior to PEI in terms of OS, CR, and LR for patients with early-stage nonsurgical HCC, which are consistent with previous studies [24,41-47]. However, in the Asian studies included in the present analysis, carcinomas near vessels were treated only with PEI, and not by RFA, which can lead to a bad prognosis. The resulting selection bias likely influenced the apparent superiority of RFA, as evidenced by the lack of difference between the techniques in the European studies. Although pooled data reported by Weis *et al.* [45] indicated that RFA is superior to PEI, their sub-analysis also showed no evidence for prolonged OS in European patients treated with RFA compared with PEI. The findings of this meta-analysis indicate that there is insufficient evidence to conclude that RFA is superior to PEI in patients with HCC tumors ≤5 cm, consistent with the results of a systematic review of the same trials [48]. Moreover, the effect of both treatments is similar for patients with small HCC tumors (<2 cm), as demonstrated by studies from Cho *et al.* [14] and Germani *et al.* [44].

There are several potential factors affecting the discrepancy between our overall findings on overall survival and previous analyses. First, race may be a latent factor that requires consideration and should not be ignored. We failed to determine the impact of race because of the shortage of required evidence. Second, the proportion of patients with Child-Pugh class B cirrhosis was higher in the two European studies than in the Asian studies. This suggests that patients with Child-Pugh A liver function treated with RFA have better outcomes, as indicated by findings reported by Brunello *et al.* [30] and Giorgio *et al.* [31]. Third, the limited number of smaller-scope studies evaluating RFA gives rise to selection bias. Fourth, the location of tumors can influence outcomes, as Bruix and Sherman [23] stated that RFA can increase the risk of severe complications in tumors located at high-risk sites. Giorgio *et al.* [31] reported that tumors located in the seventh and second liver segments are more suitable for treatment by PEI than by RFA. Similarly, Ebara *et al.* [49] found that 25% of lesions could not be treated by RFA because of the unfavorable location of the tumor. The exclusion criteria in two Asian studies in this meta-analysis included tumors located within 5 to 10 mm of the liver hila or the common bile duct [25-27], which are associated with a risk of injury to the major bile duct following RFA as reported by Brunello *et al.* [30] and Giorgio *et al.* [31]. Fifth, there is a lack of long-term data on patient survival; the RCTs that evaluated the two percutaneous treatments were small and had a short follow-up period. We found that the OS in both groups became more similar as the follow-up period lengthened, consistent with findings reported by Shen *et al.* [43] and Bouza *et al.* [24]. This is a critical factor that was not considered in previous meta-analyses [41,42,47]. Sixth, only four of the included trials reported the pathologic stage of the tumors, which were unclear and may have induced a bias effect in our study.

Similar results were found for LR and CR in the comparison between RFA and PEI. Both the total pooled data and subgroup meta-analysis results of the Asian studies implied that RFA is superior to PEI, consistent with the results of five other meta-analyses [24,41-44]. However, these findings may also be unreliable for the reasons listed above. The results of the European and African studies indicated no differences in LR at 1 year, but a significant discrepancy was present at 3 years [31]. Very similar results were obtained by Giorgio *et al.* [16]. Such findings suggest that RFA provides local tumor control and more CR than does PEI when the follow-up time is prolonged, but additional studies are needed to more thoroughly evaluate this.

We did not pool the total number of complications among all patients in the RFA and PEI groups. However, the total number of adverse events found in this meta-analysis suggests that RFA may be associated with a higher rate of adverse events, as reported by other studies [24,41-44]. This may be explained by the fact that the diameter of the RFA electrode needle is larger than that of the PEI needle, potentially leading to a higher risk of complications such as hemothorax. On the other hand, the temperature may quickly diffuse to a larger region during the performance of RFA. Temperature diffusion is difficult to control and may readily lead to complications such as skin burns. Therefore, for small HCC, PEI is associated with fewer complications and a broader scope of treatment indications than RFA.

### Limitations of this study

We acknowledge that the conclusions of this meta-analysis are limited by various factors. First, a small number of relevant studies were included. This may have led to false-negative or false-positive conclusions. In particular, only two European trials compared the OS and LR. Although the sensitivity analysis did not change the significance of the results, the outcomes of the European trials are still weakened. Second, only five studies presented survival curves or HR-related data. Third, some trials did not involve a randomization procedure, which may have affected the accuracy of the outcomes [22,36]. Third, none of the trials blinded the treatment providers because of the nature of the interventional operation. Fortunately, the primary outcome was not affected by blinding because the studies reported the mortality rates [50]. Fourth, however, the secondary outcomes may have been affected by performance and detection bias, which may have resulted in imprecise findings of this meta-analysis. Fifth, the baseline characteristics of the included studies, such as tumor size, number of lesions, and different technical procedures, may have limited the accuracy of the pooled data. Sixth, some data were directly extracted from the OS and LR curves because of the lack of original data. Although we did our best to extract and calculate the necessary data using the software Engauge Digitizer [36], some error is inevitable. Nevertheless, the Cochrane Collaborative Group recommends that not all data need to be perfectly accurate when some patient data are lacking [34]. Seventh, none of the trials were powerful enough to identify differences in OS because of small sample sizes and short follow-up durations. Finally, heterogeneity was found in the studies included in this meta-analysis. This may be explained by the fact that more patients had hepatitis B infection, higher α-fetoprotein levels, and the inclusion of HCC nodules of >5 cm in the study by Lencioni *et al.* [40]. However, technologic disparity among the studies may the most important reason for the observed heterogeneity.

A number of credible techniques were used to reduce potential bias, including an extensive search of the literature, strict guidelines regarding duplicate data extraction, the use of clear criteria, and the use of a random effects model for effect estimation and contacting the corresponding authors by email. In spite of all the above-described limitations, this study provides the most thorough comparison of RFA and PEI.

## Conclusion

The findings of the present meta-analysis indicate that there is no reliable evidence to support the idea that RFA is superior to PEI for patients, especially in Europe, with cirrhotic HCC. Additional large-scale RCTs with less selection bias are still needed.
